# Association Between Prior Selective Laser Trabeculoplasty and Phaco‐iStent Inject Efficacy

**DOI:** 10.1111/ceo.14588

**Published:** 2025-07-23

**Authors:** Jeremy C. K. Tan, Colin I. Clement, Andrew White, Hamish Dunn, Bernardo Soares, David Wechsler, Vincent Lee, Mitchell Lawlor, the FGB registry

**Affiliations:** ^1^ Save Sight Institute, Faculty of Medicine and Health, University of Sydney Sydney New South Wales Australia; ^2^ Department of Ophthalmology Prince of Wales Hospital Randwick New South Wales Australia; ^3^ Centre for Vision Research, Westmead Institute for Medical Research Westmead New South Wales Australia; ^4^ Glaucoma Investigation and Research Unit, Royal Victorian eye and Ear Hospital Centre of Eye Research Australia Melbourne Victoria Australia; ^5^ Faculty of Medicine Health and Human Sciences, Macquarie University Sydney New South Wales Australia; ^6^ South West Eye Surgery Warrnambool Victoria Australia; ^7^ Department of Glaucoma Sydney Eye Hospital Sydney New South Wales Australia

**Keywords:** iStent inject, minimally‐invasive glaucoma surgery, SLT

## Abstract

**Background:**

To evaluate the association between prior selective laser trabeculoplasty (SLT) on intraocular pressure (IOP) lowering efficacy of phacoemulsification combined with iStent inject (phaco‐iStent).

**Methods:**

Retrospective study of 1550 eyes of 1023 patients with and without prior SLT that underwent phaco‐iStent. Surgical success was defined at 12 months using three different endpoints: (A) IOP ≤ 21 mmHg and ≥ 20% IOP decrease, or ≥ 1 medication reduction vs. baseline, (B) ≥ 1 medication reduction with no increase in baseline IOP or (C) IOP ≤ 21 mmHg and ≥ 20% IOP decrease alone with no increase in baseline medication use. The prior SLT and no SLT patients were propensity score matched by age, baseline IOP and IOP‐lowering medication use and visual field mean deviation (VF MD).

**Results:**

Three hundred and sixty‐eight eyes (184 eyes per group) of 313 patients were matched based on baseline IOP and medications, VF MD, and age at surgery. Prior SLT was associated with an increased risk of failure using endpoints A [hazards ratio (HR) 2.13, 95% confidence intervals (CI) 1.34—3.38], B (HR 1.73, CI 1.1—2.72) and C (HR 1.51, CI 1.05—2.16). In eyes with prior SLT, higher baseline IOP was significantly associated with a lower risk of failure to achieve a minimum 20% reduction in IOP from baseline (HR 0.89, 0.85—0.94, *p* < 0.001).

**Conclusion:**

In this observational study, prior SLT was associated with an increased risk of failure after phaco‐iStent inject surgery. In eyes with prior SLT, there was a significantly decreased risk of failure in eyes with higher baseline IOP.

**Abbreviations**
IOP: intraocular pressure
iStent: iStent inject
MD: mean deviation
MIGS: minimally‐invasive glaucoma surgery
SLT: selective laser trabeculoplasty
VF: visual field

## 1 Introduction

Intraocular pressure (IOP) reduction in glaucoma is usually achieved by topical IOP‐lowering medications and laser therapy initially, with filtration surgery such as trabeculectomy or aqueous shunt surgery reserved for patients with more advanced disease or where IOP remains uncontrolled. Over half of patients placed on topical medications are however nonadherent within 3–4 years of treatment [1], arising from factors such as cost of therapy, side effects and forgetfulness [2]. Minimally invasive glaucoma surgery (MIGS) refers to a new class of devices which shunt aqueous humour from the anterior chamber into Schlemm's canal, suprachoroidal space, or subconjunctival space [3], and generally offers a better safety profile and faster recovery compared to filtration surgery [4, 5, 6]. This has consequently seen a growing adoption of MIGS procedures worldwide in recent years [4]. The iStent inject device (Glaukos Corporation, San Clemente, CA, USA) is a trabecular micro‐bypass stent, which is the most commonly performed MIGS procedure in the USA [4], and has been widely demonstrated to provide effective lowering of IOP alone or in combination with phacoemulsification [7, 8, 9, 10]. Selective laser trabeculoplasty (SLT) has been shown to be a cost‐effective primary treatment in open‐angle glaucoma and ocular hypertension [11]. The procedure typically uses a 532‐nm frequency‐doubled Q‐switched Nd:YAG laser with a fixed spot size of 400 μm and a pulse duration of 3 ns, delivering power levels between 0.3 and 2.0 mJ [12]. SLT reduces IOP by enhancing outflow through the trabecular meshwork (TM) via mechanisms such as structural changes, inflammatory remodelling of the extracellular matrix, and stimulation of TM cell proliferation [12].

Studies evaluating the influence of prior SLT on angle‐based MIGS efficacy outcomes have yielded conflicting results. This study evaluates the association of prior SLT on the IOP‐lowering efficacy of phacoemulsification combined with iStent‐inject (phaco‐iStent) in a large multi‐centre cohort using MIGS‐specific success endpoints.

## 2 Methods

We performed a retrospective analysis of data from the FGB registry, an audit and research tool developed to facilitate accurate and comprehensive collection of data relevant to routine clinical care. Detailed methodology of the registry has been previously described [13, 14]. Ethics approval was provided by the Royal Australian and New Zealand College of Ophthalmology Human Research Ethics Committee with reference number 2020/ETH02676. All participating centres adhered to ethical guidelines and regulations. Inclusion criteria were consecutive adult patients who underwent combined phacoemulsification and iStent inject (phaco‐iStent) implantation with at least 12 months of follow‐up data available. Patients with prior filtration surgery were excluded.

### 2.1 Primary and Secondary Outcomes

The primary efficacy endpoints were defined at 12 months as per the American Academy of Ophthalmology (AAO) Glaucoma Preferred Practice Pattern Panel guidelines [15], which allowed for either a reduction in IOP or medication use compared to baseline:

- IOP ≤ 21 mmHg and ≥ 20% IOP decrease with no increase in baseline medications, OR

≥ 1 IOP‐lowering medication reduction with no increase in baseline IOP (IOP or medication reduction)

B ≥ 1 IOP‐lowering medication reduction with no increase in baseline IOP (medication reduction)
C IOP ≤ 21 mmHg and ≥ 20% IOP decrease with no increase in baseline medications (IOP reduction)

Further criteria for success were no secondary IOP‐lowering procedure including laser or glaucoma surgery and no symptomatic hypotony (IOP < 6 mmHg with loss of ≥ 10 VA letters).

Baseline IOP refers to the IOP before phaco‐iStent surgery. No pre‐operative IOP‐lowering medication washout was performed as this was a real‐world observational study.

The secondary efficacy endpoints were rates of complete (no medications) and qualified (with medications) success defined as an upper IOP level ≤ 15, 18, or 21 mmHg and ≥ 20% decrease in IOP from baseline at 12 months, as per the World Glaucoma Association (WGA) guidelines [16]. Failure was defined as IOP/medication outcomes falling outside of the respective success definition for two consecutive visits, secondary IOP‐lowering procedure, or symptomatic hypotony. We also reported mean change in IOP (absolute and percentage) and number of medications at 12 months compared to baseline.

Prior SLT can be recorded in the FGB registry in two ways. As many surgeons use FGB to audit their outcomes, some patients will only enter FGB around the time of the phaco‐iStent inject procedure which is being audited. In this case, the baseline patient visit captures ‘prior procedures’, which is where SLT will be recorded. In this case, the time since the prior SLT is not captured. The second way it is recorded is in existing patients in the registry. Here SLT is recorded as a procedure, and then phaco‐iStent inject will be recorded as a subsequent procedure at the time of surgery. In this case, the duration of time between SLT and phaco‐iStent is available. In this cohort, all eyes with prior SLT had the procedure before the time of enrolment in the registry; therefore, time from SLT was not available.

### 2.2 Study Cohorts and Analysis

From the overall cohort of eyes that had received phaco‐iStent in the registry, we performed propensity score matching of baseline IOP, number of medications, visual field mean deviation (VF MD), and age at surgery to evaluate outcomes in a cohort of eyes with and without prior SLT with similar pre‐operative characteristics. Propensity score matching was conducted using the *MatchIt* package in R with nearest neighbour matching (1:1 ratio) based on Mahalanobis distance.

Kaplan Meier survival curves were used to compare the cumulative proportion of failure between prior SLT versus no prior SLT groups. We used multivariable mixed effects Cox regression models to compare time to failure across efficacy endpoints in prior versus no prior SLT groups. Eyes were clustered at the patient and surgeon level to account for the non‐independence of observations in bilateral cases and success bias induced by the surgeon. Models were adjusted for baseline IOP and number of medications, baseline VF MD, and age at surgery. Finally, we examined the effect of baseline IOP on failure across the primary endpoints in only eyes that had undergone prior SLT. All analyses were conducted using R (R Foundation for Statistical Computing, Vienna, Austria).

## 3 Results

One thousand five hundred fifty eyes of 1023 patients had undergone phaco‐iStent with a minimum of 12 months of follow up in the registry, which comprised 232 (15.0%) eyes that had undergone previous SLT. The baseline characteristics, primary and secondary outcomes of the original cohort of 1550 eyes are described in Tables S1 and S2. Three hundred and sixty‐eight eyes (184 eyes per group) of 313 patients were then propensity score matched based on baseline IOP and medications, VF MD, and age at surgery. There was no significant difference in baseline demographic or ophthalmic characteristics between groups; baseline IOP was 17.1 (SD 5.2) and 16.8 (SD 5.1) mmHg in the prior SLT and no prior SLT groups respectively (*p* = 0.59). Baseline number of medications was 2 (SD 1.2) in both groups (Table 1).

**TABLE 1:** Baseline characteristics of the propensity score matched cohort (*N* = 368 eyes) comprising 184 eyes with prior SLT versus no prior SLT in each group.

Category	Level	Matched cohort		
		SLT	No SLT	*p*
Eyes		184	184	
Patients		142	171	
Gender		60.60%	50.90%	0.093
Age		73.6 (7.5)	73.8 (7.2)	0.809
BCVA		74 (13.1)	75.2 (8.3)	0.278
CCT		528.5 (36.4)	535.9 (40.1)	0.063
Baseline IOP		17.1 (5.2)	16.8 (5.1)	0.586
Baseline Medications		2 (1.2)	2 (1.2)	0.913
VF MD		−5.5 (5.6)	−5.3 (5.2)	0.706
Diagnosis	Angle closure	0	1 (0.5%)	0.082
	Normal tension glaucoma	34 (18.5%)	25 (13.6%)	
	Ocular hypertension	8 (4.3%)	14 (7.6%)	
	Open angle glaucoma suspect	5 (2.7%)	13 (7.1%)	
	Primary open angle glaucoma	129 (70.1%)	117 (63.6%)	
	Secondary open angle glaucoma	8 (4.3%)	14 (7.6%)	

### 3.1 Primary and Secondary Outcomes

The primary and secondary outcomes are shown in Table 2. There was a significantly lower proportion of eyes that met the primary endpoint success criteria in the prior SLT versus no prior SLT group in the matched (47.8% vs. 60.3%, *p* = 0.021) cohort at 12 months. There was likewise a significant difference in the proportion of eyes that achieved at least 1 medication reduction (30.4 vs. 41.3%, *p* = 0.039), IOP ≤ 21 mmHg and at least 20% decrease from baseline (29.9 vs. 41.3%, *p* = 0.029) and IOP combined with medication reduction (12.5 vs. 22.3%, *p* = 0.019) in the prior SLT versus no prior SLT groups respectively. There was a significantly higher number of post‐operative IOP‐lowering medications used (1.3 vs. 1.0, *p* = 0.017), and a significant difference in the change in medication used versus baseline (−0.7 vs. −1.0, *p* = 0.013) in the prior SLT versus no prior SLT group. There was no instance of numerical or symptomatic hypotony in either group over the 12 months.

**TABLE 2:** Primary and secondary outcomes of the matched cohort (*N* = 368 eyes) at 12 months. The primary efficacy endpoints are IOP ≤ 21 mmHg and ≥ 20% IOP decrease or ≥ 1 medication decrease compared to baseline, medication reduction alone, or IOP reduction alone. The secondary efficacy endpoints are qualified and complete success, reported at the 15‐, 18‐ and 21 mmHg thresholds.

	Matched cohort		
	SLT	No SLT	*p*
Eyes	184	184	
IOP outcomes			
Baseline IOP	17.1 (5.2)	16.8 (5.1)	0.586
Final IOP	14.7 (5.2)	13.8 (3.8)	0.061
IOP Change	−2.4 (−3.3, −1.6)	−3 (−3.7, −2.3)	0.286
IOP Change percentage	−13.4% (−26, 0)	−16.4% (−33.3, 0)	0.304
Medication outcomes			
Baseline medications	2 (1.2)	2 (1.2)	0.913
Final medications	1.3 (1.4)	1 (1.2)	0.017
Medications change	−0.7 (−0.9, −0.6)	−1 (−1.2, −0.9)	0.013
Primary endpoints			
IOP ≤ 21 mmHg + ≥ 20% IOP decrease OR ≥ 1 med decrease	88 (47.8%)	111 (60.3%)	0.021
≥ 1 med decrease	56 (30.4%)	76 (41.3%)	0.039
IOP ≤ 21 mmHg + ≥ 20% IOP decrease	55 (29.9%)	76 (41.3%)	0.029
IOP ≤ 21 mmHg + ≥ 20% IOP decrease AND ≥ 1 med decrease	23 (12.5%)	41 (22.3%)	0.019
Secondary endpoint: Qualified success			
15 mmHg	59 (32.1%)	71 (38.6%)	0.23
18 mmHg	70 (38%)	80 (43.5%)	0.34
21 mmHg	72 (39.1%)	83 (45.1%)	0.291
Secondary endpoint: Complete success			
15 mmHg	25 (13.6%)	36 (19.6%)	0.161
18 mmHg	27 (14.7%)	40 (21.7%)	0.105
21 mmHg	28 (15.2%)	40 (21.7%)	0.14

### 3.2 Time to Failure Across Primary Endpoints

**FIGURE 1:**
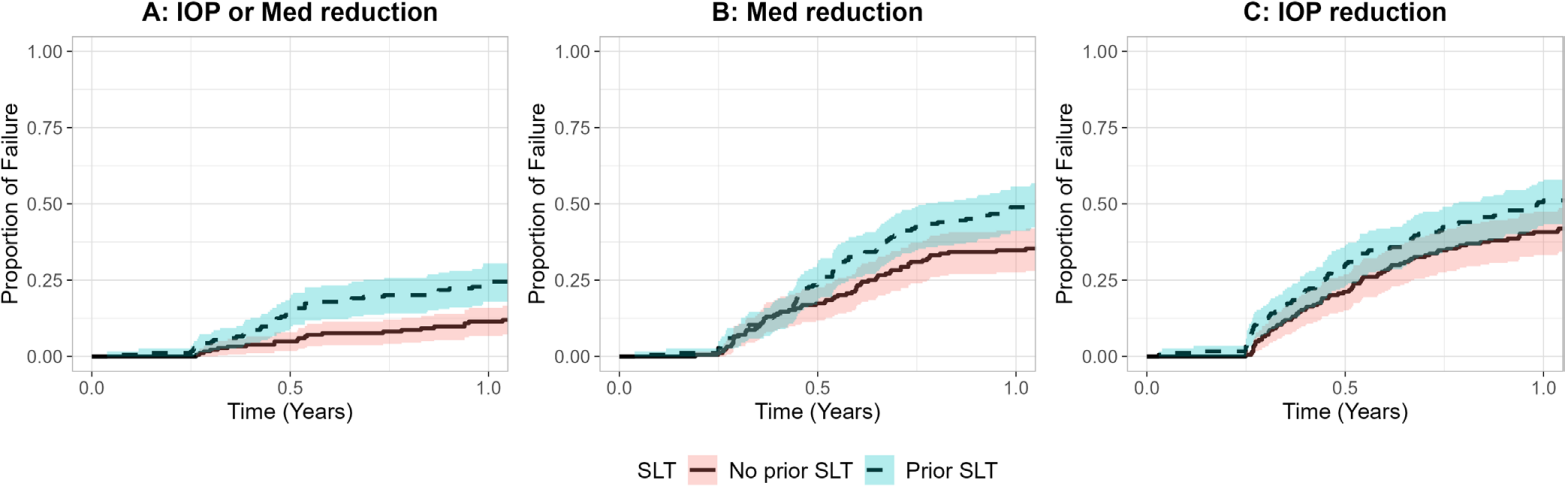
Kaplan–Meier curves of proportion of failure following phaco‐iStent inject surgery up to 1 year post‐operatively in the matched cohort (*N* = 368) in eyes with prior SLT (dashed line) versus no prior SLT (solid line) when using the following definitions: (A) IOP ≤ 21 mmHg and ≥ 20% IOP decrease or ≥ 1 medication reduction vs. baseline (no washout), (B) ≥ 1 medication reduction with no increase in baseline IOP, (C) IOP ≤ 21 mmHg and ≥ 20% IOP decrease alone with no increase in baseline medication. There was a statistically significant increase in failure across the three definitions.

Figure 1 shows the Kaplan Meier survival curves of the proportion of eyes that failed the primary endpoint of IOP ≤ 21 mmHg and ≥ 20% IOP decrease or ≥ 1 medication reduction vs. baseline (Figure 1A), 1 medication reduction with no increase in baseline IOP (Figure 1B) and IOP ≤ 21 mmHg and ≥ 20% IOP decrease alone with no increase in baseline medication (Figure 1C) in the matched cohort. In the multivariable mixed effects Cox models, prior SLT was associated with a significantly increased risk of failure to achieve an IOP or medication reduction (HR 2.13, 1.34–3.38, *p* = 0.001), a medication reduction (HR 1.73, 1.1—2.72, *p* = 0.017), or an IOP reduction (HR 1.51, 1.05–2.16, *p* = 0.025) (Table 3). Baseline IOP and medications were observed to have significant associations with failure across the primary endpoints. The Kaplan Meier survival curves in the overall cohort are shown in Figure S1.

**TABLE 3:** Coefficients, confidence intervals and P values for multivariable mixed effects Cox regression models and primary efficacy endpoints in the matched cohort (*N* = 368 eyes). The endpoints are: (A) IOP ≤ 21 mmHg + ≥ 20% IOP decrease or ≥ 1 medication reduction vs. baseline, (B) ≥ 1 medication reduction with no increase in baseline IOP and (C) IOP ≤ 21 mmHg + ≥ 20% IOP decrease alone with no increase in baseline medication.

	Matched					
	A: IOP or med reduction		B: Med reduction		C: IOP reduction	
	Coefficient (CI)	*p*	Coefficient (CI)	*p*	Coefficient (CI)	*p*
Prior SLT	2.13 (1.34–3.38)	0.001	1.73 (1.1–2.72)	0.017	1.51 (1.05–2.16)	0.025
Baseline IOP	1 (0.96–1.05)	0.903	1.07 (1.03–1.11)	0.001	0.87 (0.83–0.9)	< 0.001
Baseline Meds	0.85 (0.69–1.05)	0.127	0.73 (0.6–0.88)	0.001	1.22 (1.04–1.43)	0.014
Age	0.97 (0.94–1)	0.095	0.99 (0.96–1.02)	0.637	0.99 (0.97–1.02)	0.494
Baseline VF MD	0.97 (0.93–1.02)	0.228	0.98 (0.95–1.02)	0.391	1 (0.97–1.03)	0.958

### 3.3 Baseline IOP and Time to Failure in Eyes With Prior SLT

We performed a sub‐analysis of the association between baseline IOP and medications and failure in only eyes that had undergone prior SLT in the overall cohort (*N* = 232 eyes). Higher baseline IOP was significantly associated with a lower risk of failure to achieve a minimum 20% reduction in IOP from baseline (HR 0.89, 0.85—0.94, *p* < 0.001). There was, however, no significant association between baseline IOP and failure to achieve an IOP or medication reduction (endpoint A) or medication reduction alone (endpoint B, Table S3). Figure S2 shows the Kaplan Meier survival curves of the proportion of eyes that failed the primary endpoint of ≥ 20% IOP decrease or ≥ 1 medication reduction versus baseline, 1 medication reduction with no increase in baseline IOP and ≥ 20% IOP decrease alone with no increase in baseline medication with baseline IOP divided into three strata: < 15 mmHg, > 15–18 mmHg and > 18 mmHg. There was no significant association between baseline medications and failure to achieve any of the three primary efficacy endpoints (Table S1). Figure S3 shows the proportion of failure across the three endpoints, with baseline medications divided into three strata: 0 or 1 medication, 2 medications, or ≥ 3 medications.

## 4 Discussion

In this study we evaluated the association between prior SLT and IOP‐lowering outcomes of phaco‐iStent inject in a large multicentre cohort of eyes. We found that prior SLT was significantly associated with a higher risk of failure at 12 months. In eyes with prior SLT, there was a significant decrease in the risk of failure in eyes with higher baseline IOP.

### 4.1 Defining Success Using MIGS‐Specific Endpoints

The WGA consensus guidelines on definitions of surgical success using IOP as a surrogate end‐point were published to address a lack of consistency in the reporting of glaucoma surgical trial outcomes [16]. These guidelines comprise the reporting of IOP success with multiple upper thresholds and one lower limit (e.g., 6 mmHg), a minimum percentage reduction in IOP and specifying complete versus qualified success. While these guidelines are useful in clinical trials that include washout of medication at baseline, they may be problematic in cases when there is no washout, which is characteristic of most observational studies. In addition, angle‐based MIGS devices tend to display a modest reduction in IOP [17, 18] while clinicians may also choose to use them in eyes with baseline IOP already within the normal range, to decrease IOP‐lowering medication use. The reduction in medication use as an efficacy endpoint is currently not adequately captured by the WGA success criteria. The AAO Glaucoma Preferred Practice Pattern Panel recently recommended the definition of surgical success for MIGS combined with phacoemulsification [15]. In this study we used the latter success definition to capture the IOP reduction expected in angle‐based MIGS, which allowed for either an IOP or medication reduction.

### 4.2 Association of SLT With Phaco‐iStent Efficacy

Reports on the association of prior SLT on angle‐based MIGS outcomes in the literature are limited and conflicting. Maier et al. evaluated the efficacy of standalone iStent inject surgery in 66 eyes with open angle glaucoma with and without prior SLT [19]. The authors found no significant difference in the decrease in IOP reduction at 12 months in both groups (19.8 vs. 19.0%). Klamann et al. reported the outcomes of Trabectome surgery in 74 patients with and without prior SLT within 3 months pre‐operatively [20]. The authors also found no significant difference in IOP between eyes with prior versus no prior SLT at all post‐operative timepoints up to 6 months in eyes with POAG [20]. In eyes with PXFG and PDG however, eyes with prior SLT had significantly lower IOP at 6 months, although this may be confounded by the higher baseline IOP in the no‐SLT group [20].

Mitchell et al. reported the outcomes of four angle‐based MIGS procedures—Canaloplasty, Goniotomy, Trabectome, and iStent (G1) with and without prior argon or selective laser trabeculoplasty (LTP) from the IRIS registry [21]. The authors used propensity score matching to evaluate outcomes of 954 eyes that underwent standalone MIGS and 7522 eyes that underwent combined phaco‐MIGS from the registry. They found that eyes with prior LTP in both standalone and combined cohorts had a significantly higher risk of failure over 36 months compared to eyes without prior LTP. While these findings are informative and parallel our findings, the primary failure endpoint was subsequent glaucoma reoperation, and as such does not adequately assess failure according to established consensus guidelines. Indeed, only 139 (9.0%) eyes in our overall cohort underwent a secondary glaucoma procedure, of which 91 (5.9%) eyes underwent laser trabeculoplasty. This contrasted with a significantly higher rate of failure across the primary MIGS‐specific IOP and medication endpoints as observed in Figure 1. Our findings therefore indicate that eyes with prior SLT may have a significantly higher risk of failure to achieve either a meaningful IOP or medication reduction compared to baseline, compared to those without SLT.

### 4.3 Possible Mechanisms of Increased Failure in Prior SLT

We observed an association between prior SLT and poorer phaco‐iStent efficacy in our cohort. Our findings may, however, be confounded by several inherent limitations of observational studies. Firstly, selection bias is an important confounder that could explain the greater risk of failure in eyes with prior SLT. The latter may be a surrogate marker for eyes with more advanced disease and with a longer history of glaucoma, with the laser procedure performed at an earlier timepoint of their treatment journey. IOP‐lowering eyedrops may have been subsequently re‐introduced following inadequate IOP reduction. Alternatively, some clinicians may use SLT as a non‐surgical alternative to patients who are progressing in an effort to avoid surgery. This, in turn, would enrich the prior SLT cohort with patients at a later point of their treatment journey, and who are at higher risk of progression. We therefore sought to mitigate these factors by analysing a cohort with matched baseline characteristics. Secondly, while SLT and the iStent inject implant may have different mechanisms of action, they likely share common downstream pathways to increase aqueous outflow. SLT employs the principle of selective thermolysis, using radiation energy to target pigmented cells in the TM without causing significant thermal damage to surrounding tissues [12]. While the mechanism by which SLT lowers IOP is not well understood, it is postulated that the laser induces multiple biologic and biochemical changes in the TM, which allows for greater aqueous outflow [12]. The iStent inject implant conversely lowers IOP by acting as a channel between the anterior chamber and Schlemm's canal, allowing aqueous to bypass the site of highest resistance—the juxtacanalicular TM [22]. Eyes with previous SLT may already have increased aqueous drainage through structures downstream to the TM, which would limit the benefit gained from implanting an iStent. Thirdly, SLT may cause some structural damage to the TM and Schlemm's canal [23], such as an inflammatory response in the TM and downstream structures. This may affect the functionality of the iStent inject implant in improving physiologic aqueous drainage.

### 4.4 Limitations and Clinical Implications

While observational data from clinical registries are an important source of real‐world evidence [24], another important limitation is that treatment decisions are not protocol driven. For instance, the decision to undertake phaco‐iStent surgery could be for reasons aside from uncontrolled IOP or the desire to reduce medication use. Post‐operatively, the decision to continue, cease, or re‐start IOP‐lowering medications is also at each clinician's discretion and influenced by numerous factors including the target IOP and disease severity. The primary endpoints we have chosen are also based on IOP reduction, which may not fully capture treatment efficacy in reducing visual field progression [25, 26]. Different clinician preferences may in turn affect success rates. We however attempt to accommodate different approaches with our primary endpoints by allowing for either an IOP or medication reduction compared to baseline. Secondly, in eyes with prior SLT, we did not have information on the duration of time between SLT and phaco‐iStent surgery, efficacy of SLT, and time to failure after SLT. These variables, especially the absence of data on the interval between prior SLT and phaco‐iStent surgery is a key limitation, as this timing may significantly influence surgical response. Finally, the confirmation of positioning of the trabecular devices intra‐operatively and post‐operatively was not documented; these devices are small and may not have been implanted in the correct location, which may affect their IOP‐lowering efficacy [27]. It is not clear that the rate of malposition is likely to be different between the two cohorts assessed here. Anterior‐segment optical coherence tomography imaging may be useful in correlating microstructure with surgical outcomes [28, 29, 30], with associations found between iStent device positioning and IOP‐lowering efficacy [31, 32]. We also accounted for the bias induced by surgeon technique and difference in outcomes across surgeons using random effects in our Cox models.

While our findings demonstrate a negative association between prior SLT and phaco‐iStent efficacy, future large prospective studies are required to investigate this in greater detail. This association is important in guiding patient selection for angle‐based MIGS to optimise success, including from a health economics perspective. Our findings may nevertheless be useful in guarding prognosis and aiding in clinical decision‐making by providing insights into which patients are most likely to benefit from angle‐based MIGS.

### 4.5 Conclusion

Prior SLT may be associated with an increased risk of failure after phaco‐iStent inject surgery. In eyes with prior SLT, there was a significant decrease in the risk of failure in eyes with higher baseline IOP.

## Conflicts of Interest

J.T. has received honoraria from Glaukos to present part of this work at the United Kingdom and Eire Glaucoma Society conference 2024. Nil other relevant conflicts of interest for remaining authors.

## Supporting information


Data S1.

## Data Availability

The data that support the findings of this study are available on request from the corresponding author. The data are not publicly available due to privacy or ethical restrictions.
