# Supplementary material for: Association Between Prior Selective Laser Trabeculoplasty and Phaco‐iStent Inject Efficacy
Source: Clin Exp Ophthalmol. 2025 Jul 23;53(9):1148–55. doi: 10.1111/ceo.14588 (PMC12747488; doi:10.1111/ceo.14588)
Supplement: Supplementary file 1 — Data S1. [file CEO-53-1148-s001.pdf]

Supplementary material

Supplementary table 1: Baseline characteristics of overall cohort (N = 1550 eyes) comprising eyes with prior SLT versus no prior SLT.

		Overall cohort		
Category	Level	SLT	No SLT	P value
Eyes		232	1318	
Patients		175	864	
Gender		60%	53.50%	0.074
Age		72.9 (7.3)	74 (7.7)	0.044
BCVA		74.2 (12.4)	73.8 (11.4)	0.648
CCT		530.8 (37.4)	540.7 (39.1)	<0.001
Baseline IOP		16.7 (5.2)	16 (4.6)	0.056
Baseline Medications		2 (1.2)	1.6 (1.1)	<0.001
VF MD		-5.5 (5.5)	-4.4 (5)	0.019
Diagnosis	Angle Closure	0	15 (1.1%)	
	Normal Tension Glaucoma	40 (17.2%)	157 (11.9%)	
	Ocular Hypertension	11 (4.7%)	176 (13.4%)	
	Open Angle Glaucoma Suspect	5 (2.2%)	117 (8.9%)	
	Primary Open Angle Glaucoma	166 (71.6%)	767 (58.2%)	
	Secondary Open Angle Glaucoma	10 (4.3%)	85 (6.4%)	

Supplementary table 2: Primary and secondary outcomes of the overall cohort (N = 1550 eyes) at 12 months. The primary efficacy endpoints are a ≥20% IOP decrease and IOP ≤ 21mmHg or ≥ 1 medication decrease compared to baseline. The secondary efficacy endpoints are Qualified and Complete success, reported at the 15-, 18- and 21 mmHg thresholds.

	Overall cohort		
	SLT	No SLT	P value
Eyes	232	1318	
**IOP outcomes**			
Baseline IOP	16.7 (5.2)	16 (4.6)	0.056
Final IOP	14.3 (5)	13.7 (3.8)	0.082
IOP Change	-2.4 (-3.1, -1.7)	-2.3 (-2.6, -2)	0.797
IOP Change percentage	-13.9% (-26.8, 0)	-13% (-28.7, 2.9)	0.709
**Medication outcomes**			

Baseline medications	2 (1.2)	1.6 (1.1)	<0.001
Final medications	1.3 (1.4)	0.7 (1)	<0.001
Medications change	-0.7 (-0.9, -0.6)	-0.9 (-0.9, -0.8)	0.079
**Primary endpoints**			
IOP ≤ 21mmHg + ≥20% IOP decrease OR ≥ 1 med decrease	108 (46.6%)	694 (52.7%)	0.026
≥ 1 med decrease	69 (29.7%)	493 (37.4%)	0.01
IOP ≤ 21mmHg + ≥20% IOP decrease	69 (29.7%)	445 (33.8%)	0.137
IOP ≤ 21mmHg + ≥20% IOP decrease AND ≥ 1 med decrease	30 (12.9%)	244 (18.5%)	0.029
**Secondary endpoint: Qualified Success**			
15 mmHg	76 (32.8%)	431 (32.7%)	0.945
18 mmHg	89 (38.4%)	478 (36.3%)	0.812
21 mmHg	92 (39.7%)	486 (36.9%)	0.679
**Secondary endpoint: Complete Success**			
15 mmHg	34 (14.7%)	261 (19.8%)	0.059
18 mmHg	36 (15.5%)	292 (22.2%)	0.016
21 mmHg	37 (15.9%)	297 (22.5%)	0.018

Supplementary table 3: coefficients, confidence intervals and P values for multivariable mixed effects cox regression models and primary efficacy endpoints in eyes with prior SLT (N = 232). The endpoints are: (A) ≥20% IOP decrease or ≥ 1 medication reduction vs baseline, (B) ≥ 1 medication reduction with no increase in baseline IOP and (C) ≥20% IOP decrease alone with no increase in baseline medication.

	A: IOP or Med reduction		B: Med reduction		C: IOP reduction	
Term	Coefficient	P value	Coefficient	P value	Coefficient	P value
Baseline IOP	0.99 (0.93 - 1.05)	0.792	1.06 (1 - 1.13)	0.06	0.89 (0.85 - 0.94)	<0.001
Baseline Medications	0.89 (0.68 - 1.16)	0.374	0.9 (0.67 - 1.22)	0.507	1.2 (0.98 - 1.47)	0.073
Age	0.99 (0.95 - 1.03)	0.64	0.99 (0.94 - 1.04)	0.714	1 (0.97 - 1.03)	0.826
Baseline VF MD	0.97 (0.92 - 1.02)	0.17	0.99 (0.93 - 1.04)	0.648	0.99 (0.96 - 1.03)	0.795

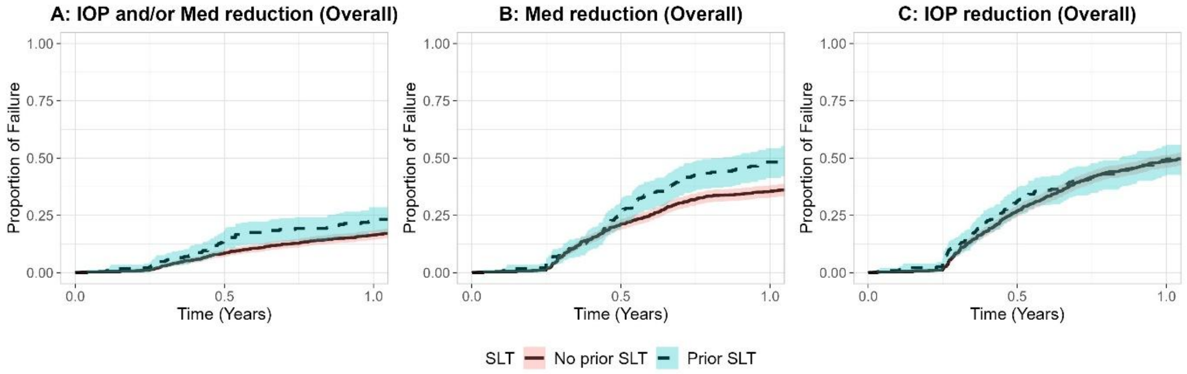

Supplementary figure 1: Kaplan-Meier curves of proportion of failure following phaco-iStent inject surgery up to one year post-operatively in the overall cohort in eyes with prior SLT versus no prior SLT (N = 1550 eyes) when using the following definitions: (A) IOP ≤ 21 mmHg and ≥20% IOP decrease or ≥ 1 medication reduction vs baseline (no washout), (B) ≥ 1 medication reduction with no increase in baseline IOP, (C) IOP ≤ 21 mmHg and ≥20% IOP decrease alone with no increase in baseline medication. There was a statistically significant increase in failure across the three definitions.

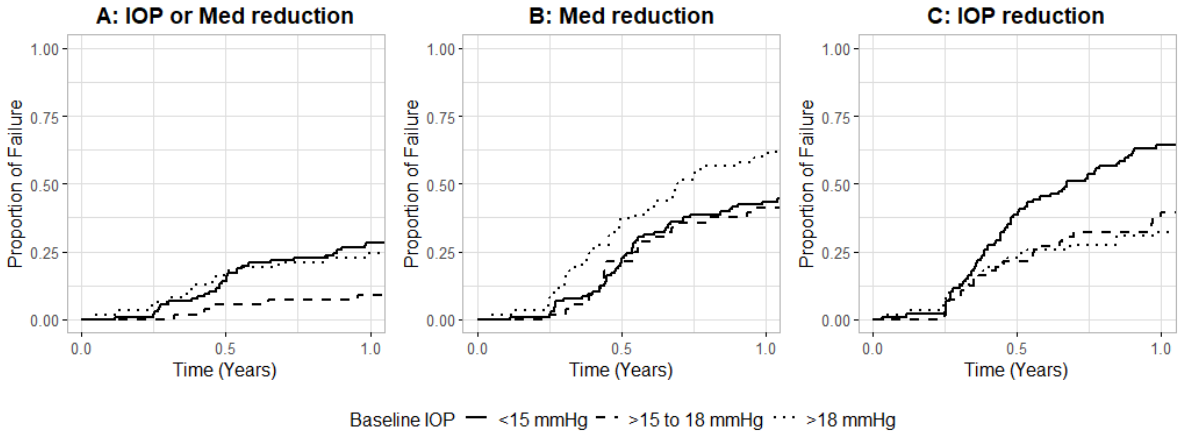

Supplementary figure 2: Proportion of failure following phaco-iStent inject surgery up to one year post-operatively in eyes with prior SLT only, across strata of baseline IOP (< 15 mmHg, >15 to 18 mmHg and > 18 mmHg). The endpoints are: (A) ≥20% IOP decrease or ≥ 1 medication reduction vs baseline, (B) ≥ 1 medication reduction with no increase in baseline IOP and (C) ≥20% IOP decrease alone with no increase in baseline medication. Confidence intervals are not shown for clarity of visualization.

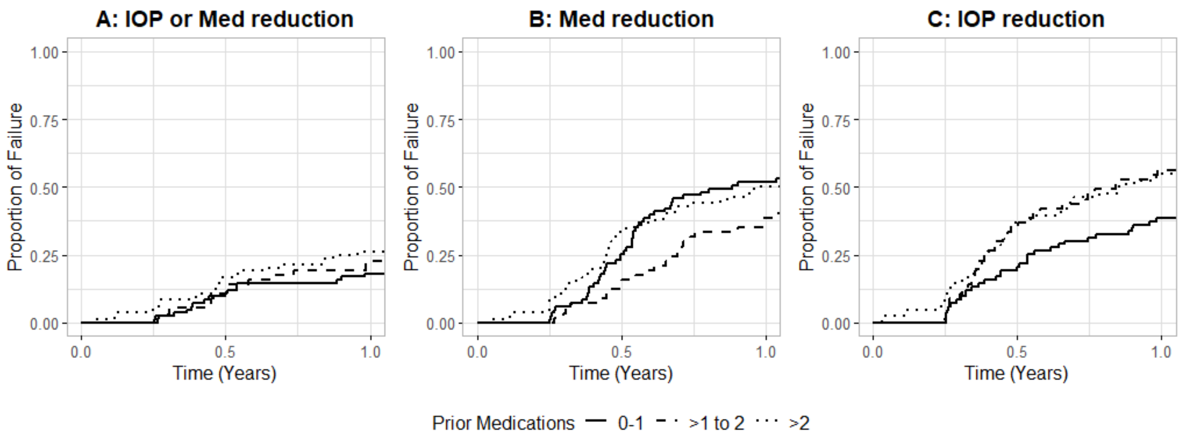

Supplementary figure 3: Proportion of failure following phaco-iStent inject surgery up to one year post-operatively in eyes with prior SLT only, across strata of baseline medications (0 or 1, 2 and > 3). The endpoints are: (A) ≥20% IOP decrease or ≥ 1 medication reduction vs baseline, (B) ≥ 1 medication reduction with no increase in baseline IOP and (C) ≥20% IOP decrease alone with no increase in baseline medication. Confidence intervals are not shown for clarity of visualization.
